# The Prognostic Value of Platelet‐Albumin‐Bilirubin Score in Patients Undergoing Transcatheter Aortic Valve Replacement

**DOI:** 10.1002/clc.70119

**Published:** 2025-03-18

**Authors:** Farman Ullah Khan, Muhammad Ammar ul hassan khan, Muhammad Aamir khan, Shad Khan, Muhammad Ismail

**Affiliations:** ^1^ Ayub Medical College Abbottabbad Pakistan; ^2^ Khyber Medical College Peshawar Pakistan; ^3^ Gomal Medical College D.I.Khan Pakistan; ^4^ Jinnah Medical College Peshawar Pakistan

**Keywords:** Platelet‐Albumin‐Bilirubin Score, transcatheter aortic valve replacement

Dear Editor,

We read with great interest the article “The Prognostic Value of Platelet‐Albumin‐Bilirubin Score in Patients Undergoing Transcatheter Aortic Valve Replacement.” by Duan et al. [1]. Reading such a well‐written and complete piece is satisfying and the author's efforts on this important subject must be acknowledged. This study comprehensively evaluates the predictive efficacy of the platelet‐albumin‐bilirubin (PALBI) score for mortality in patients having transcatheter aortic valve replacement (TAVR), as well as its significance for enhancing risk classification alongside the Society of Thoracic Surgeons (STS) score. Although the research provides significant insights, some aspects demand further consideration and discussion.

First of all, this study only used the PALBI score, which includes platelet count, albumin, and bilirubin, in assessing liver function in TAVR patients. However, it did not consider other liver prognostic models, such as the Child‐Pugh (CP) and Model for End‐Stage Liver Disease (MELD) scores, which involve biomarkers such as creatinine and INR. A multicenter study found that using various hepatic prognostic models can improve outcome prediction in TAVR patients [2].

Additionally, this investigation excluded significant comorbidities that have reduced the PALBI score's predictive validity, including frailty, systemic inflammation, malnutrition, and anticoagulant treatment. These factors significantly affect albumin and platelet counts, which could reduce PALBI's precision as a predictor. By incorporating these variables into multivariate models, PALBI's feasibility as an independent predictor of TAVR‐related mortality would be strengthened. The 2018 study found that comorbid conditions like malnutrition significantly affect TAVR outcomes. In TAVR patients, low serum albumin, a gauge of both malnutrition and frailty, has been associated with a higher 30‐day mortality rate and more postoperative complications [3].

Furthermore, the PALBI score may not provide a complete risk assessment for patients undergoing TAVR because it does not account for key cardiac‐specific factors such as left ventricular ejection fraction (LVEF), aortic valve gradient (AVG), or the presence of coronary artery disease. Relying only on the PALBI score ignoring these key cardiac characteristics may result in an inadequate assessment of patient risk, thus limiting its predictive accuracy in TAVR populations. Reduced LVEF and low AVG are substantially related with poor post‐TAVR outcomes, with low AVG providing as a significant predictor of mortality [4].

This study does not include people with chronic liver illness, end‐stage renal disease (ESRD), and dialysis, limiting its credibility. These high‐risk patients typically have poor post‐TAVR outcomes, their exclusion may underestimate PALBI's true prognostic value in real‐world circumstances. Chronic kidney disease (CKD) and ESRD have a considerable impact on TAVR outcomes, with studies indicating that dialysis‐dependent patients have higher mortality rates and a higher 1‐year mortality than non‐dialysis patients [5]. Including these patients in future study would allow for a more thorough assessment of PALBI's prediction accuracy across a wide spectrum of TAVR groups.

In spite of these limitations, the study offers a useful viewpoint on the PALBI score's predictive function in TAVR risk comparison. Validation of its therapeutic relevance requires future studies with bigger, multi‐center cohorts, thorough risk modeling, and inclusion of high‐risk individuals. The authors' efforts to improve risk stratification in TAVR patients are much appreciated.

## Conflicts of Interest

The authors declare no conflicts of interest.

## Data Availability

The authors have nothing to report.
